# Recent Progress in Fabrications and Applications of Heating-Induced Long Period Fiber Gratings

**DOI:** 10.3390/s19204473

**Published:** 2019-10-15

**Authors:** Cailing Fu, Yiping Wang, Shen Liu, Zhiyong Bai, Changrui Liao, Jun He, Ying Wang

**Affiliations:** 1 Guangdong and Hong Kong Joint Research Centre for Optical Fibre Sensors, College of Physics and Optoelectronic Engineering, Shenzhen University, Shenzhen 518060, China; fucailing89@163.com (C.F.); shenliu@szu.edu.cn (S.L.); baizhiyong@szu.edu.cn (Z.B.); cliao@szu.edu.cn (C.L.); hejun07@szu.edu.cn (J.H.); yingwang@szu.edu.cn (Y.W.); 2 Key Laboratory of Optoelectronic Devices and Systems of Ministry of Education and Guangdong Province, Shenzhen University, Shenzhen 518060, China

**Keywords:** long period fiber gratings, fiber optic sensors, orbital angular momentum, mode converter

## Abstract

This paper presents a review of our work concerning the recent progress in fabrications and applications of heating-induced long period fiber gratings (LPFGs). Firstly, three kinds of heating fabrication techniques based on CO_2_ laser, hydrogen–oxygen flame and arc discharge are demonstrated to fabricate LPFGs, i.e., standard LPFGs (SLPFGs) and helical LPFGs (HLPFGs), in different types of optical fibers such as conventional fibers, photonic crystal fibers, and photonic bandgap fibers. Secondly, the all-fiber orbital angular momentum (OAM) mode converters based on heating-induced SLPFGs and HLPFGs in different types of fibers are studied to increase the transmission capacity. Finally, the heating-induced SLPFGs and HLPFGs are investigated to develop various LPFG-based strain, pressure, torsion and biochemical sensors.

## 1. Introduction

The optical fiber gratings play a vital role in the field of optical communications and fiber optical sensing. In-fiber gratings are divided into two types, i.e., fiber Bragg gratings (FBGs) with periodicities of the order of the optical wavelength [1,2,3,4] and long period fiber gratings (LPFGs) with periodicities of several hundred wavelengths [5,6,7]. The working principle of the LPFG is the coupling between the forward-propagating core mode and several cladding modes co-propagating in the same direction. Vengsarkar et al. wrote the first standard LPFG (SLPFG) with periodic refractive index change in the conventional glass fiber core using an ultraviolet (UV) laser radiation in 1996 [8]. Compared with SLPFG, helical LPFG (HLPFG) refers to a fiber where there exists a periodical helical structure such as a screw index-modulation along the fiber axis [9,10,11,12,13,14,15,16,17,18,19,20,21,22]. A HLPFG with double helix symmetry was firstly produced by twisting glass optical fiber with a noncircular core cross section as it passed through a miniature oven [9]. Following this, the fabrications and applications of LPFGs, including SLPFGs and HLPGs, were rapidly developed. Various methods to fabricate the LPFG, such as CO_2_ laser [23,24,25,26,27,28,29,30,31,32], arc discharge [33,34,35,36,37,38], hydrogen–oxygen flame heating [13,14,15,16,17,22], femtosecond laser [39,40], mechanical micro-bend [41,42], etched corrugations [43,44], and ion beam implantation [45], have been proposed and demonstrated. One of the key steps of fabricating the LPFG is to introduce periodic refractive index modulation in the fiber. Among above-mentioned fabrication techniques, CO_2_ laser, arc discharge, and hydrogen–oxygen flame all heat the fiber into a fused status, then the periodic refractive index modulation in the fiber is reserved due to the possible mechanisms, i.e., residual stress relaxation, physical deformation, and structural changes. Various heating-induced LPFGs in conventional fiber [12,13,22,28,35,46,47], photonic crystal fiber (PCF) [14,15,16,29,48], and photonic bandgap fiber (PBF) [27,30,31] could then be successfully written. LPFG-based devices have recently attracted great attention and found versatile applications, such as in all-fiber orbital angular momentum (OAM) mode converters [11,13,14,17,49,50,51,52,53,54,55,56], and strain [48,57,58,59,60,61,62,63,64], pressure [16,29,30,65,66], torsion [16,19,38,46] and biochemical sensors [67,68,69,70,71,72,73].

This paper presents a review of our recent work concerning the recent progress in fabrications and applications of heating-induced LPFGs. The heating fabrication techniques, i.e., CO_2_ laser, arc discharge, and hydrogen–oxygen flame heating, for writing SLPFGs and LPFGs in conventional glass fiber, PCF and PBF are presented in Section 2. Then the in-fiber orbital angular momentum (OAM) mode converters based on SLPFGs and HLPFGs are described in Section 3. Subsequently, the strain, pressure, torsion and biochemical sensors using the heating-induced LPFGs are outlined in Section 4, Section 5, Section 6 and Section 7, respectively. Finally, the review paper is concluded.

## 2. Fabrications

Various fabrication techniques have been demonstrated and improved to fabricate high-quality SLPFGs and HLPFGs in different types of optical fibers. Compared with SLPFG, HLPFG refers to a fiber where there exists a periodical helical structure such as a screw index-modulation along the fiber axis [9,10,11,12,47]. The heating fabrication techniques, i.e., CO_2_ laser, arc discharge, and hydrogen–oxygen flame as the heat source, could be used to write SLPFGs and HLPFGs in conventional glass fiber, PCF, and PBF. 

### 2.1. CO_2_ Laser Heating Techniques

Since Davis et al. reported the first CO_2_-laser-induced SLPFG in a conventional glass fiber [6,7], various CO_2_ laser heating techniques have been demonstrated and improved to obtain high-quality SLPFGs. A typical point-to-point CO_2_-laser irradiation technique is periodically moving the fiber along its axis direction. Rao et al. demonstrated a CO_2_-laser fabrication system in which an industrial two-dimensional optical scanner was employed to write high-quality SLPFGs with nearly zero insertion loss [23,24,25]. However, a lower power stability of the industrial CO_2_ laser leads to a poor reproducibility of the fabricated SLPFGs.

**Figure 1:**
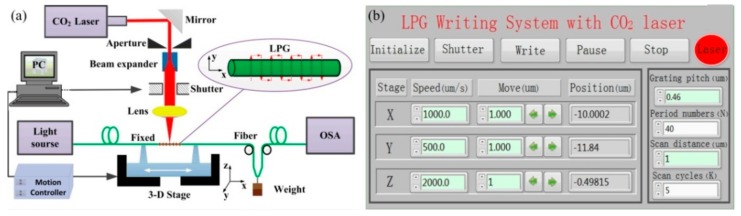
(**a**) Experimental setup and (**b**) operation interface of the standard long period fiber grating (SLPFG) fabrication system by using CO_2_ laser heating technique [28].

As shown in Figure 1, the authors demonstrated a SLPFG fabrication system, consisting of an industrial CO_2_ laser (SYNRAD 48-1), an electric shutter, a ZNSE PO/CX lens, a four-times beam expander, and a three-dimensional stage, based on an improved two-dimensional scanning technique by use of a focused CO_2_ laser [28,29,31,32,48,74]. An improved stability and reproducibility of the fabrication system is attributed to the employed closed loop control system. The SLPFG fabricating process could be started, paused and stopped randomly by using the buttons “Write”, “Pause”, and “Stop”, respectively [28]. As shown in Figure 1a, firstly, one end of a fiber is fixed by a dual-arm fiber holder on the three-dimensional motorized stage and another end is fixed by a small weight, providing a constant pre-strain to enhance the efficiency of inscribing SLPFGs. Secondly, the fiber is heated across the “Y” direction using the CO_2_ laser, thus creating the first period. Thirdly, the stage is shifted along the “X” direction with the set grating pitch (Λ), then repeating step 2. These steps are repeated until the last period, i.e., repeating N times. Finally, the above-mentioned process is repeated for K cycles until a desired SLPFG is created.

**Figure 2:**
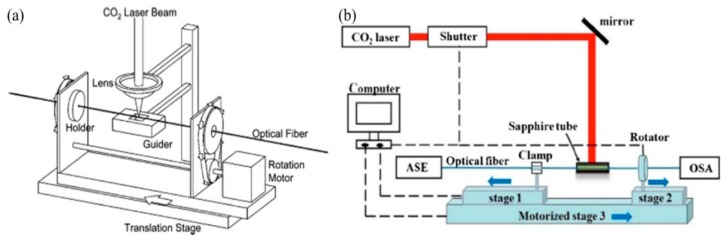
(**a**,**b**) Experimental setups for fabricating the helical LPFG (HLPFG) by use of CO_2_ laser [10,11].

In addition, the HLPFG fabrication systems by use of CO_2_ laser heating technique are also demonstrated. As shown in Figure 2a, the experimental setup for fabricating the HLPFG is composed of a rotation motor, a translation stage, two fiber holders, a focal lens, and a guider by means of CO_2_ laser [10]. While the fiber is rotated by use of the rotation motor, the actuator translates the fiber along its axis. The helical refractive index modulation is induced by CO_2_ laser beam irradiation onto a fiber while it rotates and moves continuously along the optical fiber axis. The HLPFG showed a similar transmission spectrum to that of a SLPFG [10]. 

The large power fluctuations of the CO_2_ laser led to the poor reproducibility of the HLPFGs. To solve this problem, Wang et al. added a sapphire tube to heat the fiber uniformly [11]. As shown in Figure 2b, CO_2_ laser heating technique is experimentally improved by using the sapphire tube to take place of the focal lens. The sapphire tube is used to provide a stable and constant temperature inside to heat the fiber to the fused status. The mechanism of the formed HLPFGs could be explained as the inherent core-cladding eccentricity during fiber drawing [11].

**Figure 3:**
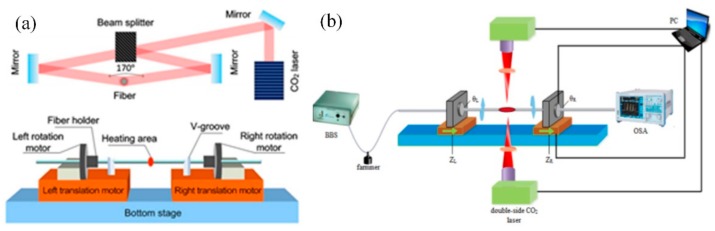
(**a**,**b**) Schematic diagrams for fabricating HLPFG using double-side CO_2_ laser [12,47].

As shown in Figure 3a, a commercial fusion splicer (Fujikura, LAZERMaster LZM-100) using the CO_2_ laser as a heat source was developed to fabricate HLPFG. The CO_2_ laser incorporated with a real-time feedback system was divided into two beams to heat the fiber in two contrary directions [12]. Compared with Figure 2b, a smaller beam size of the CO_2_ laser would ensure a smaller fused area to be twisted with the rotation motor correctly, increasing the accuracy. As shown in Figure 3b, Li et al. designed a double-side CO_2_ laser fabrication system. Firstly, the fiber was twisted with 180° in the opposite directions by the two rotator motors. Then, the double-side CO_2_ laser was employed to irradiate the fiber [47].

Using these inscribing systems based on CO_2_ laser, various types of heating-induced SLPFG and HLPFG in conventional glass fiber, PCF and PBF are fabricated.

#### 2.1.1. LPFGs in Conventional Fiber

**Figure 4:**
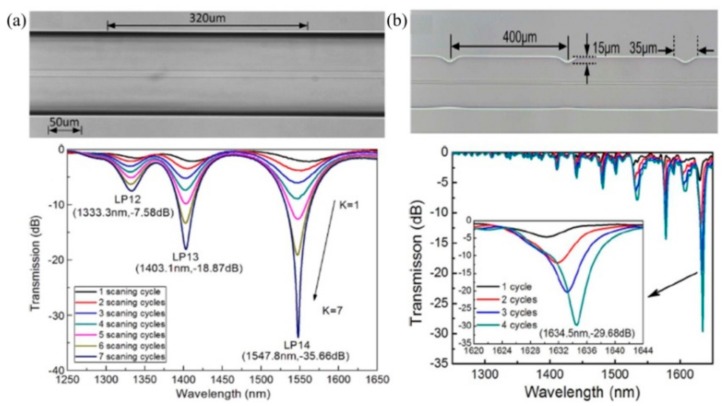
Microscope image and transmission spectrum of the CO_2_-laser-inscribed SLPFGs in (**a**) single mode fiber (SMF) [28] and (**b**) thin core fiber (TCF) [32].

As shown in Figure 4a, one high-quality heating-induced SLPFG in a standard single mode fiber (SMF) with a dip attenuation of –35.66 dB and a low insertion loss of less than 0.3 dB was achieved by using the experimental setup in Figure 1 [28]. During the fabrication, the resonant wavelength shifted toward the shorter wavelength, and the attenuation increased with the increase of K. No obvious deformation on the surface was observed, and possible mechanisms for refractive index modulation in the SLPFGs are the glass densification and residual stress relaxation [74].

Then, an asymmetrical SLPFG was also inscribed in a thin core fiber (TCF) [32]. A local high temperature resulting from the repeated scanning of the CO_2_ laser created a local high temperature in the TCF, which led to the melting and gasification of SiO_2_ on the fiber surface. Consequently, periodic grooves with a notch depth and notch width of 15 and 35 µm, respectively, were carved on one side of the TCF, as shown in Figure 4b. The resonant wavelength shifted toward a longer wavelength while K increased, which is opposite to that of the SLPFGs in SMF [28]. Such heating-induced SLPFG exhibited a narrowed 3-dB bandwidth and high polarization-dependent loss of 8.7 nm and 20 dB. The large polarization-dependent loss resulted from the single-side exposure of the CO_2_ laser. Moreover, the author also successfully inscribed a SLPFG in a four-mode fiber (FMF). Such a SLPFG could be used as a OAM mode converter, i.e., generating OAM_±1_ modes [49].

**Figure 5:**
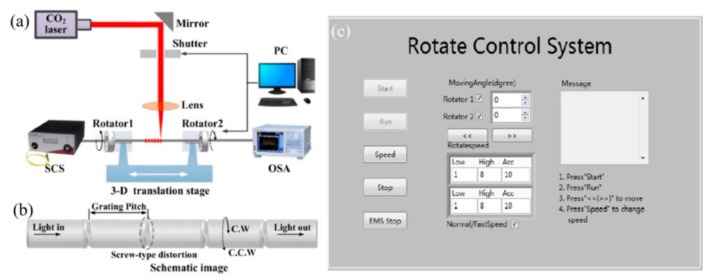
(**a**) Schematic diagram for fabricating pre-twisted LPFG by use of CO2 laser; (**b**) microscope image of a right-helix pre-twisted LPFG with permanent screw-type deformation; (**c**) operation interface of the pre-twisted LPFG fabrication system [46].

A new type of CO_2_-laser-induced LPFG with a helical structure has been proposed and demonstrated. The authors fabricated a pre-twisted LPFG with permanent screw-type deformations by periodically twisting a SMF under CO_2_ laser irradiation [46]. The fabrication system for inscribing a pre-twisted LPFG was built by improving the experimental setup in [28]. As shown in Figure 5a, rotator1 and rotator2 were employed to simultaneously twist the fused SMF in opposite directions to form the screw-type deformations in the fiber. As show in Figure 5c, a LabVIEW program was completed to control the rotation angle (β), velocity (ν) and direction, where the pre-twist of the LPFG is calculated using the equation α=2β/L. The schematic of the pre-twisted LPFG is illustrated in Figure 5b, which could be used as a torsion sensor with enhanced sensitivity.

Moreover, Oh et al. presented an optical torque sensor based on a HLPFG in SMF by the experimental setup in Figure 2a [10]. Subsequently, three kinds of thinned HLPFGs with smaller diameters were also fabricated by the improved experimental setup in Figure 2b [11], and then a flat-top band-rejection filter was obtained by successively cascading two HLPFGs with opposite helicities [75].

In addition to the HLPFGs in SMF, the HLPFG in two-mode fiber (TMF) has also been fabricated using CO_2_ laser [76]. Shen et al. have demonstrated a HLPG in a multi-core fiber by means of combing the CO_2_ laser heating technology and twist process [77]. The HLPFG in multi-core fiber shows great potential as a twist sensor and can be applied for measuring twist directions. The HLPFG inscribed in polarization-maintaining fiber by CO_2_ laser heating technique has been investigated experimentally. The achieved HLPFG could be used as a PDL compensator owing to a high polarization extinction ratio of more than 30 dB at the resonant wavelength [78]. HLPFGs have recently drawn considerable attention in these applications, such as torsion sensors [16], band-rejection filter [75,79] and conversion of orbital angular momentum (OAM) modes [13,14,15,17,18,20], due to their inherent helical structure and low polarization-dependent loss. This section reviews these heating fabrication techniques.

#### 2.1.2. LPFGs in PCF

Solid-core PCFs have attracted great attention owing to their special microstructures in the cladding and optical properties. Since Eggleton et al. reported the grating in a photosensitive PCF with a Ge-doped core in 1999 [80], various LPFGs in different types of PCFs have been reported [81]. Kakarantzas et al. fabricated a structural SLPFG in pure-silica solid-core PCF via CO_2_ laser. The periodic hole-size perturbation facing the CO_2_ laser induced the core mode coupling to the cladding mode [82].

**Figure 6:**
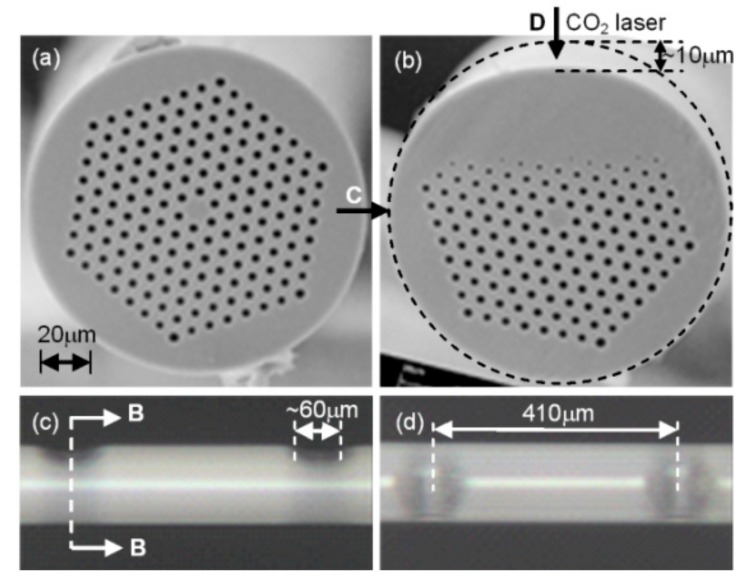
Scanning electron micrographs of the employed photonic crystal fiber (PCF) (**a**) before (**b**) after CO_2_ laser irradiation; (**c**,**d**) micrographs of the obtained asymmetrical SLPFG with periodic grooves [57].

**Figure 7:**
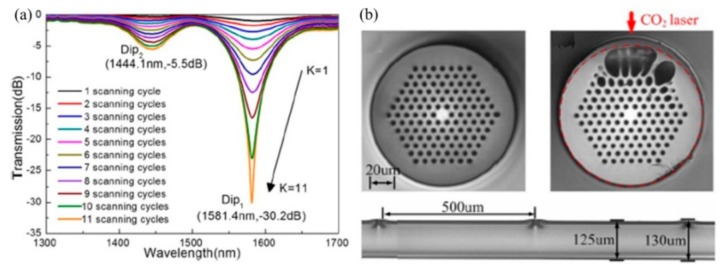
(**a**) Transmission spectrum evolution of a CO_2_-laser-heated inflated SLPFG (I-SLPFG) in a solid-core PCF while the number of scanning cycles increases; (**b**) microscope images of the I-SLPFG with periodic inflations [29,48].

As shown in Figure 6, an asymmetrical SLPFG with periodic grooves was written in a PCF by use of a focused CO_2_ laser beam [57,74]. The periodic refractive index modulations in the fiber are induced by the periodic grooves with a depth and width of 15 and 50 μm, respectively, as shown in Figure 6b, thus creating a heating-induced SLPFG in the PCF. This asymmetrical SLPFG has unique optical properties, e.g., high strain sensitivity, low temperature sensitivity and high polarization dependence. Then an inflated SLPFG (I-SLPFG) in the PCF, as shown in Figure 7, was fabricated by means of periodically inflating the air holes, i.e., a pressure-assisted CO_2_ laser heating technique [48]. When the air pressure with a value of ∼1.5 MPa in the PCF was stable, then the PCF was periodically heated. As a result, the periodic inflations of the air holes are caused by the CO_2_-laser-induced high temperature and high-pressure air, thus introducing periodic refractive index modulation in the PCF. Such periodic inflations enhanced the strain [48] and pressure sensitivity [29].

In addition to these asymmetrical SLPFGs, the symmetrical SLPFGs in PCF were also fabricated by CO_2_ laser irradiation by using a 120° gold-coated reflecting silicon mirror [26]. Furthermore, Zhu et al. reported an extremely short LPFG with eight periods and a 2.8-mm length in a PCF via a point-to-point CO_2_ laser technique, where obvious physical deformation in the fiber was observed [83]. In contrast, another strain-insensitive and high-temperature SLPFG without geometrical deformation and elongation of the PCF was fabricated by periodic mechanically residual stress due to CO_2_ laser heating [84]. The CO_2_ laser heating technique could also be used to fabricate SLPFGs in polarization-maintaining PCF. Results show that the irradiation orientation of the CO_2_ laser beam strongly influences the writing efficiency. The highest efficiency was obtained when the irradiation orientation is along the slow axis of the fiber [85].

**Figure 8:**
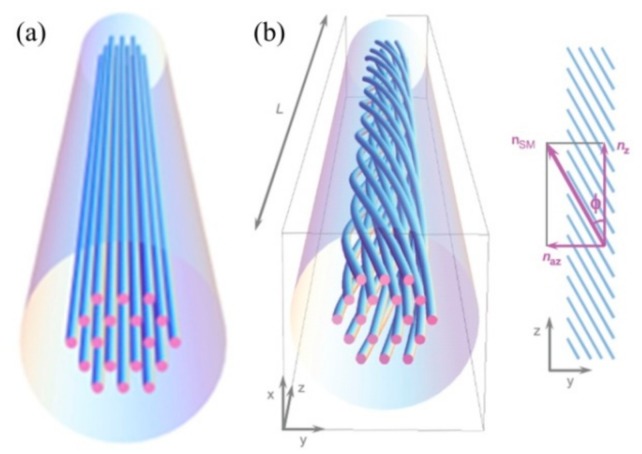
Schematics of (**a**) a solid-core PCF and (**b**) HLPFG in PCF. The blue tubes are the air-hole channels in the cladding area [18].

Compared with SLPFG in PCF, a helical PCF, i.e., a so-called HLPFG in PCF, with a series of helices for the air-hole in the cladding, was repeated by the twisted period, as illustrated in Figure 8b [18]. The periodic helical microstructure of the HLPFG in the PCF creates a series of unusual and fascinating effects. In an un-twisted PCF, the air-holes in the cladding move along the fiber axis, as illustrated in Figure 8a.To date, various methods were proposed and demonstrated to introduce the helical structure in the fiber by applying external mechanical torsion [86], spinning the preform during the fiber drawing [87] and using the heat-processing methods.

**Figure 9:**
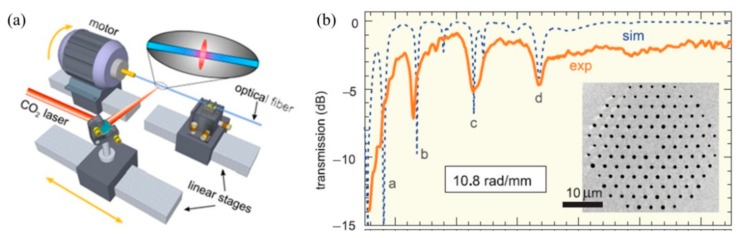
(**a**) Experimental setup for fabricating the HLPFG in PCF by use of CO_2_ laser; (**b**) the simulated and measured transmission spectrum of HLPFG in PCF [18].

As shown in Figure 9a, one end of the PCF is fixed by the fiber holder, another end is mounted at the center of a motorized rotator. As shown in Figure 8b, light in the cladding, i.e., the air-hole of the PCF, is constrained to follow a helical path due to the helical lattice of air holes in the cladding. Then, the discrete OAM resonance resulting from the azimuthal momentum could be generated, i.e., the resonance dips in the transmission spectrum [28]. The circular birefringence in the spectral regions without OAM was studied [21]. A continuous permanent twist rate was induced in the three-bladed Y-shaped core PCF by using a CO_2_ laser as heat source [20]. The twist period was a few millimeters or less, and the HLPFG have been shown to support helical Bolch waves and exhibit OAM birefringence. HLPFGs in the PCF have recently drawn considerable interest such as generation of the OAM modes, formation of the HLPFG [9,10], suppression of the high-order mode in lasers [88], and the sensors [19,89].

#### 2.1.3. LPFGs in PBF

PBF, including air-core PBF, fluid-filled PBF and all-solid PBF, confines light in a low refraction index core by photonic bandgap effect of the cladding. High order resonances between fundamental core mode and cladding super modes of LPFGs in all-solid PBF are demonstrated through a point-to-point side illumination process using CO_2_ laser [90]. Air-core PBFs have potential to overcome some of the fundamental limitations of solid fibers, promising, for example, reduced transmission loss, lower nonlinearity, higher damage thresholds and lower latency [91].

**Figure 10:**
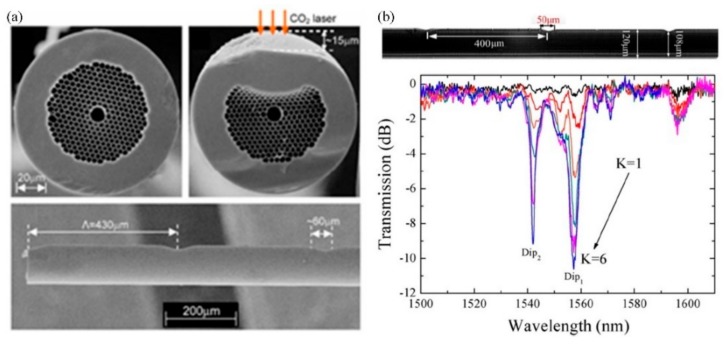
(**a**) Scanning electron micrographs of SLPFG in air-core photonic bandgap fiber with periodic notches; (**b**) transmission spectrum evolution of SLPFG while the scanning cycles increases [27,30].

Wang et al. reported a SLPFG in an air-core PBF using a similar experimental setup in [25], i.e., a focused CO_2_ laser beam, to periodically deform/perturb air holes along the fiber axis [27]. As shown in Figure 10a, the outer rings of air holes facing the CO_2_ laser are largely deformed, which is induced by the ablation of glass on the fiber surface and the partial or complete collapse of air holes in the cladding due to the CO_2_-laser-induced local high temperature. The dominant factor of the formed SLPFG in air-core fiber is the periodic geometrical deformations that causes resonant mode coupling.

The author further fabricated SLPFGs in air-core PBF using the improved experimental setup in [28], i.e., an improved two-dimensional scanning technique by use of a CO_2_ laser beam [30]. Compared with 50 scanning cycles [27], as shown in Figure 10b, a high coupling efficiency of two resonant dips, i.e., Dip_1_, Dip_2_, could be up to –10.61 dB and –9.15 dB, respectively, with only six scanning cycles, indicating that the writing efficiency was greatly improved. The relatively low power fluctuation, i.e., a power stability of ±2%, improves the uniformity of the periodic collapse along the fiber axis, enhancing the mode coupling. Hence, a SLPFG in air-core PBF with relatively large depth at the resonant dip is expected by use of fewer scanning cycles. Such an SLPFG could be used to develop a promising gas pressure sensor. Furthermore, the pressure sensitivity was effectively enhanced by use of a short hollow silica tube drilled with a micro-channel [30].

### 2.2. Hydrogen–Oxygen Flame Heating Techniques

**Figure 11:**
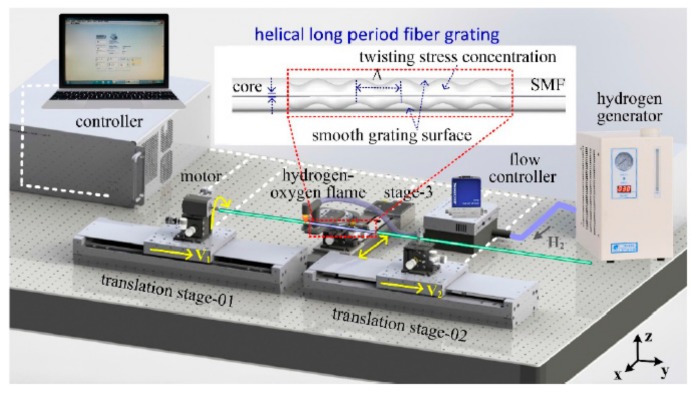
Experimental setup for fabricating the HLPFG [22].

As shown in Figure 11, the author developed a novel heat-processing technique, i.e., hydrogen–oxygen flame, to fabricate the HLPFGs. The hydrogen–oxygen flame heating system, consisting of a rotation rotator, two translation stages, and hydrogen–oxygen flame, was developed to fabricate an HLPFG. One end of the fiber was mounted on translation stage-02, another end was fixed along the rotation motor on translation stage-01. During heating, the rotation motor and two translation stages were employed to induce periodic helical structures in the employed fiber. Using this HLPFG inscription method, small numbers of HLPFGs could be obtained by twisting the fiber once. Moreover, various types of HLPFGs in conventional glass fiber [13,17,22] and solid-core PCF [14,15,16] could be fabricated.

The HLPFG inscription can be described as follows [13]. First, the employed fiber was heated into a fused status with hydrogen–oxygen flame. While the two translation stages moved synchronously with velocities of v_1_ and v_2_, the rotation motor rotated synchronously with a twist rate of Ω. Then an HLPFG with periodic helical refractive index modulation was obtained. In addition, the helical pitch (Λ), i.e., grating pitch, was given by the equation Λ = v_2_ × 60/Ω.

#### 2.2.1. LPFGs in Conventional Glass Fiber

**Figure 12:**
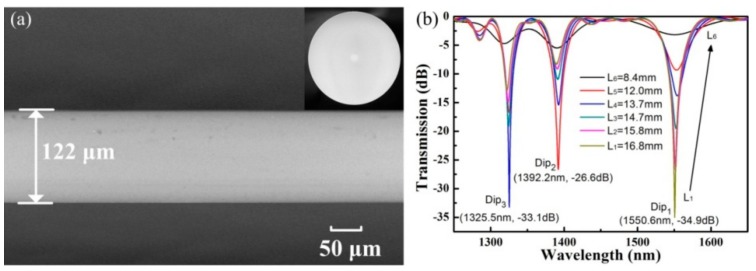
(**a**) Scanning electron micrographs and (**b**) transmission spectrum evolution of the HLPFG in SMF while the length decreases [13].

**Figure 13:**
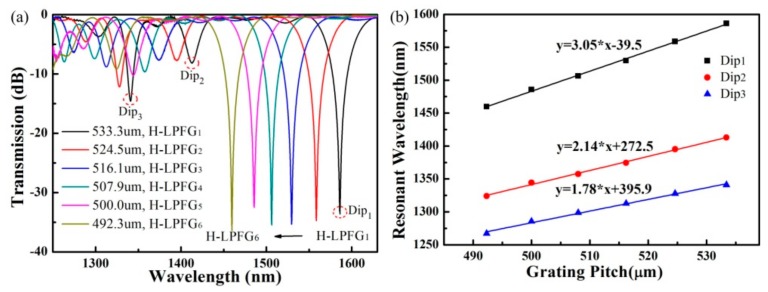
(**a**) Transmission spectra of six HLPFGs in SMF with different helical grating pitches; (**b**) measured resonant wavelength of the HLPFG versus helical grating pitch [13].

As shown in Figure 12a, no obvious physical deformation was observed in the obtained helical fiber, while the decreased diameter, i.e., 122 μm was caused by the velocity difference between v_1_ and v_2_. In the experiment, v_1_ and v_2_ were set as 1.56 and 1.60 mm/s, respectively. As shown in Figure 12b, the resonant wavelength, corresponding to Dip_1_, shifts towards a longer wavelength, and the maximum attenuation decreases from −34.9 to −3.2 dB, while the length of the helical SMF is cut from 16.8 to 8.4 mm. Then, six HLPFG samples with different grating pitches were also fabricated, as shown in Figure 13a. The stability and high-efficiency of the hydrogen–oxygen flame heating system could be reflected by the low insertion loss and strong coupling strength of the HLPFGs. The obtained HLPFG in the SMF with reserved periodic refractive modulations could add helical phase to generate OAM modes.

**Figure 14:**
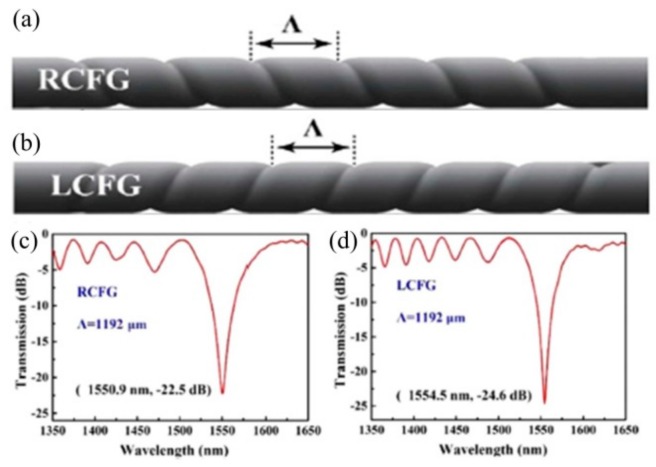
Diagram of periodic helical structure in the (**a**) right-handed and (**b**) left-handed HLPFG in four-mode fiber (FMF); Transmission spectra of (**c**) the right-handed and (**d**) left-handed HLPFG [17].

The right-handed and left-handed HLPFG in four-mode fiber (FMF) were also fabricated by applying the opposite motor directions, i.e., clockwise- or anticlockwise-twisted, respectively [17]. As shown in Figure 14, the resonant wavelength and attenuation were almost same for the righted and left-handed HLPFG in the FMF with a high coupling efficiency up to 99%. When the light propagated through the HLPFG, every period directly added a helical phase delay to the coupled modes, thus creating an OAM mode. Thus, a resonant enhancement of the helical phase can be formed in the coupled modes, that is, OAM modes.

#### 2.2.2. LPFGs in PCF

**Figure 15:**
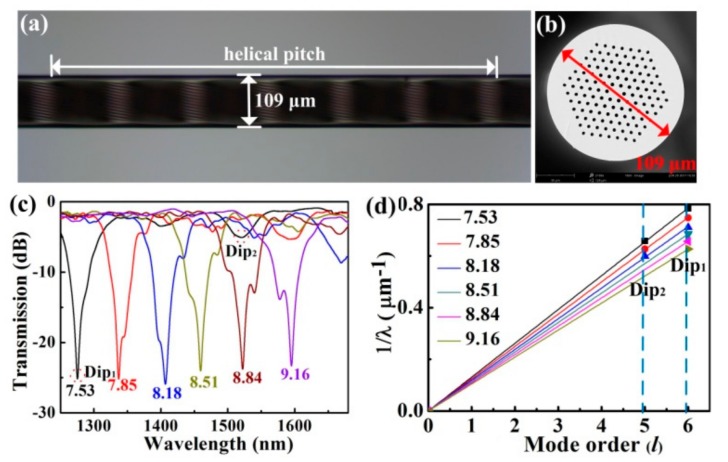
(**a**) Side-view and (**b**) cross-section microscope images of the HLPFG in PCF; (**c**) transmission spectra of six HLPFG samples with different twist rates; (**d**) reciprocal resonant wavelength (λ_R_) in units of at μm^−1^ plotted against mode order for the experimental data [14].

A novel HLPFG in a solid-core PCF was fabricated by use of the post-processing method, i.e., hydrogen–oxygen flame heating [14]. After twisting the PCF with Ω = 115 rpm, v_1_ = 1.38 mm/s, and v_2_ = 1.60 mm/s, a HLPFG was obtained, as shown in Figure 15. When the PCF is gently twisted (αΛ ≪ 1), the “space-filling” mode (SM) is constrained to forward in a helical trajectory, creating discrete OAM modes [18]. 

Six HLPFG samples with different twist rates were fabricated. As shown in Figure 15c,d the resonant wavelength (λ_R_) shifted to a longer wavelength with the increase of the twist rate. Moreover, the equation of the resonant wavelength (λ_R_) is:

(1) $$\lambda_{R}=2\pi n_{SM}\rho^{2}\alpha /\left|l\right|$$

The product n_SM_ρ^2^ is a constant for a given PCF. Then the resonance dips, i.e., Dip_1_ and Dip_2_, of the HLPFG in PCF in Figure 15 could generate OAM_+6_ and OAM_+5_ modes, respectively.

**Figure 16:**
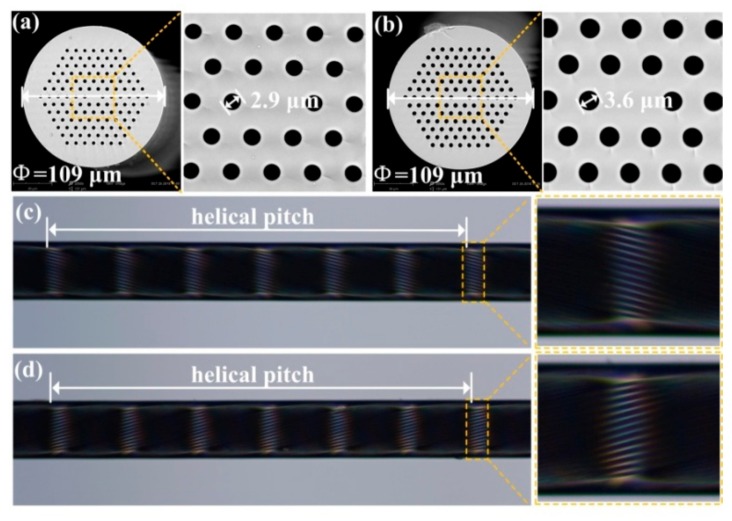
Cross-section of the (**a**) HLPFG and (**b**) inflated HLPFG (I-HLPFG) in the PCF; side-view microscope images of the (**c**) clockwise-twisted (CT)- and (**d**) anticlockwise-twisted (ACT)-IHLPFG [15].

**Figure 17:**
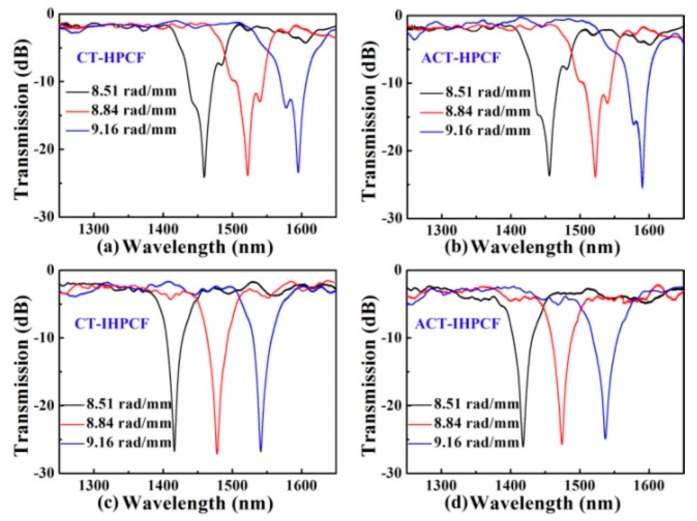
Transmission spectra for (**a**) CT-HLPFG; (**b**) ACT-HLPFG; (**c**) CT-IHLPFG; and (**d**) ACT-IHLPFG [15].

Furthermore, an improved hydrogen–oxygen flame heating system for fabricating an inflated HLPFG (I-HLPFG) in the PCF was built by adding an air pump in the experimental setup in [13]. As shown in Figure 16c,d, the clockwise-twisted (CT) and anticlockwise-twisted (ACT) IHLPFG with an opposite periodic pattern of the helical air holes on the fiber surface, were fabricated by applying opposite directions of the rotation motor [15]. As shown in Figure 17a,b, compared with un-inflated HLPFG, i.e., an air hole diameter of 2.9 μm, the air holes in IHLPFG exhibited a larger diameter, i.e., 3.6 μm, due to the inflated gas pressure of 0.8 MPa.

Compared with the IHLPFGs, as shown in Figure 17, the resonant wavelength of the HLPFGs shifts toward a longer wavelength under the same twist rate condition. Moreover, the distinct splits in the HLPFG are attributed to an asymmetric refractive index modulation. In contrast, the perfect resonant dips without splits were obtained for the IHLPFG, owing to the inflated high-pressure air avoiding the uneven shrinkage of the air holes in cladding. Thus, a perfect IHLPFG could be achieved by means of an inflation-assisted hydrogen–oxygen flame heating technique.

### 2.3. Arc Discharge Heating Techniques

Arc discharge heating techniques have drawn attention owing to their simplicity, flexibility, and thermal stability. The possible mechanisms of the LPFG formation are geometrical deformation, stress relaxation, and structure changes. 

A high-temperature stability SLPFG in S-doped and N-doped fiber was fabricated using the arc discharge technique [92]. The origin of antisymmetric perturbation of the fiber in arc-induced SLPFGs that couple the core mode into the antisymmetric LP_1i_ cladding modes is the temperature gradient in the arc discharge. This gradient causes a temperature gradient in the fiber, which results in a gradient of viscosity and a corresponding asymmetry of fiber deformation [93]. In addition, the PDL of the SLPFGs strongly depends on the arc discharge fabrication parameters (electric current, arc duration, and pulling tension) [34]. Esposito et al. has made a detailed review concerning the fabrication of SLPFGs in several fibers, i.e., silica optical fiber with both different dopants and geometrical structures, by means of arc discharge heating technique [94].

**Figure 18:**
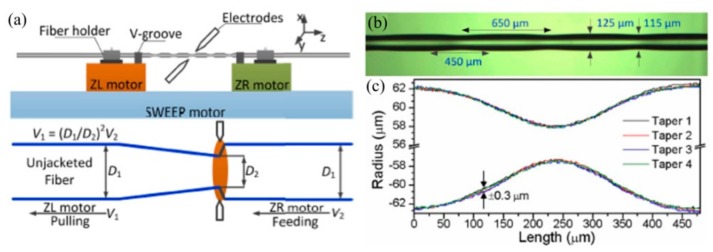
(**a**) Schematic diagram for inscribing a LPFG with periodic tapers; (**b**) side-view microscope images of periodic taper; (**c**) shape profiles of four tapers [35].

As shown in Figure 18, a commercial fusion splicer (ARC master FSM-100P+) with a second program development, consisting of three motors, i.e., ZL, ZR, and SWEEP motors, is employed to fabricate the SLPFG by periodically tapering the SMF [35]. Firstly, the down-taper and up-taper are formed by synchronously stretching the fiber with ZR and ZL motors. Secondly, the second taper pitch was realized by the SWEEP motor. The velocities of the ZL and ZR motors are v_1_ and v_2_, respectively. In addition, the pulling velocity v_1_ is determined by the equation v_1_ = (d_1_/d_2_)^2^v_2_, where d_1_ and d_2_ are the diameters of SMF and the processed taper, respectively. Therefore, the proposed arc discharge technique could be used to inscribe SLPFGs with periodic tapers [65].

**Figure 19:**
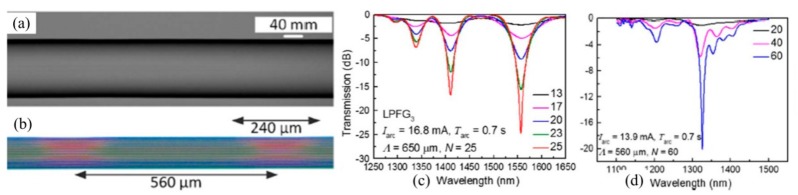
Side-view microscope images of fabricated SLPFG in (**a**) SMF and (**b**) PCF; transmission spectrum of the SLPFG in (**c**) SMF and (**d**) PCF [36].

Subsequently, Yin et al. also developed an automatic arc discharge heating technology to simultaneously fabricate the SLPFGs in the SMF and PCF [36]. As shown in Figure 19, the obtained SLPFGs sample in SMF and PCF have no obvious deformation on the surface. It takes only 25 and 60 arc discharges periods to reach the dip attenuation of 20 dB in SMF and PCF, respectively. The improved fabrication efficiency is attributed to the appropriate arc duration and current.

**Figure 20:**
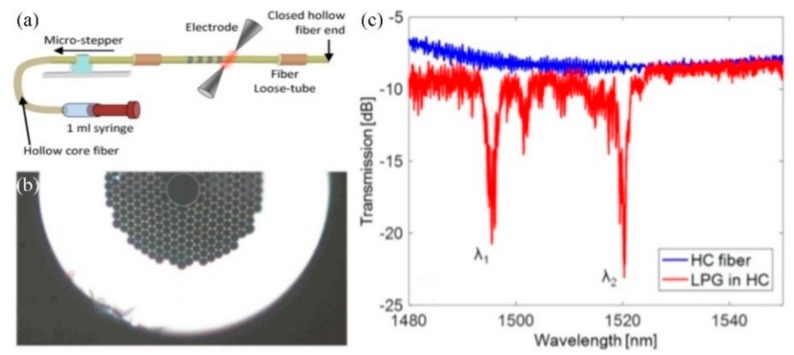
(**a**) Schematic diagram; (**b**) microscopy image; and (**c**) transmission spectra of the SLPFG in air-core PBF by using the pressure-assisted arc discharge heating technique [95].

To avoid the air holes in the cladding region collapsing, Iadicicco et al. used a modified pressure-assisted arc discharge heating technique to fabricate SLPFGs in air-core PBF, as shown in Figure 20. The combination consisted of an electric arc discharge, to locally heat the fiber, and a static pressure, to preserve the holey structure of the PBF. A static pressure was forced into the air holes by means of connecting a small air pump with the air-core PBF [95]. Subsequently, they reported about the fabrication of SLPFGs in polarization-maintaining fiber by means of arc discharge heating technique, regardless of the fiber orientation during the fabrication process. The transmission spectrum was dependent on the state of the input light polarization, i.e., the fast and slow axes of the Panda fiber [96]. 

**Figure 21:**
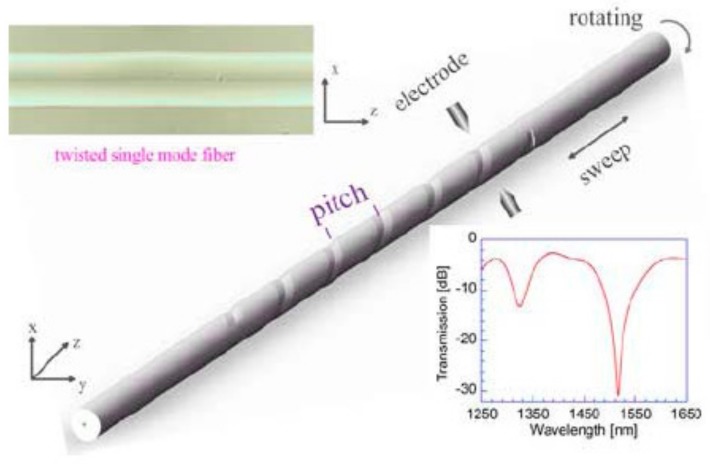
Schematic diagram of fabricated HLPFG in SMF [37].

Arc discharge heating techniques could also be employed to fabricate the HLPFG. Sun et al. employed a commercial fusion splicer, i.e., an automatic arc discharge heating technique, to inscribe high-quality HLPFGs in SMF [37], as shown in Figure 21. The HLPFG inscription program involved two motors, i.e., θ_L_ and SWEEP motors. The θ_L_ motor with the twist speed was used to determine the time for rotating a period, while the SWEEP motor was to move the fiber according to the grating pitch of the HLPFG. Then, the arc-induced screw-type refractive index modulation was formed in the HLPFGs. The achieved HLPFGs have been reported to have potential usage as sensors in temperature, refractive index, twist stress, and strain.

**Figure 22:**
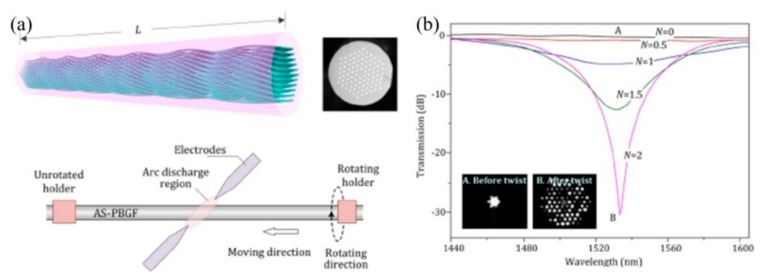
(**a**) Schematic diagrams and (**b**) transmission spectra of HLPFG in all-solid PBF using the arc discharge heating techniques [38].

Li et al. presented a high-repeatability and -stability arc discharge technique based on the secondary exploration of the splicer [38]. As shown in Figure 22, the attenuation of the resonance dip for HLPFG in an all-solid PBF reached up to 30 dB with only two periods. During the fabrication process, the appropriate arc strength, the velocity of the translation and rotation motor are vital to the quality of the HLPFG in all-solid PBF.

## 3. OAM Mode Converters

Orbital angular momentum (OAM) mode is characterized by a dark hollow center with phase or polarization singularities, which has potential applications in various fields, such as optical communications, detection of rotating objects, and optical tweezers. Achieving a higher transmission capacity of the optical communication system could be realized by using mode-division multiplexing (MDM) technique based on the generated all-fiber OAM modes. Recently, many spatial OAM mode converters have been demonstrated, such as spatial light modulators [97], phase plates [98], integrated silicon devices [99] and micrometer-scale meta-materials [100]. However, these spatial OAM mode converters have an obvious coupling loss when integrated with the existed optical fiber communication system. A key device is a mode converter, i.e., coupling from the fundamental core mode to high-order mode, which utilizes the generated OAM modes to realize the MDM. Recently, various all-fiber OAM mode converters based on SLPFGs or HLPFGs in different types of fibers have been reported, due to their low loss, high coupling efficiency and easy fabrication.

### 3.1. OAM Mode Converters Based on SLPFGs

**Figure 23:**
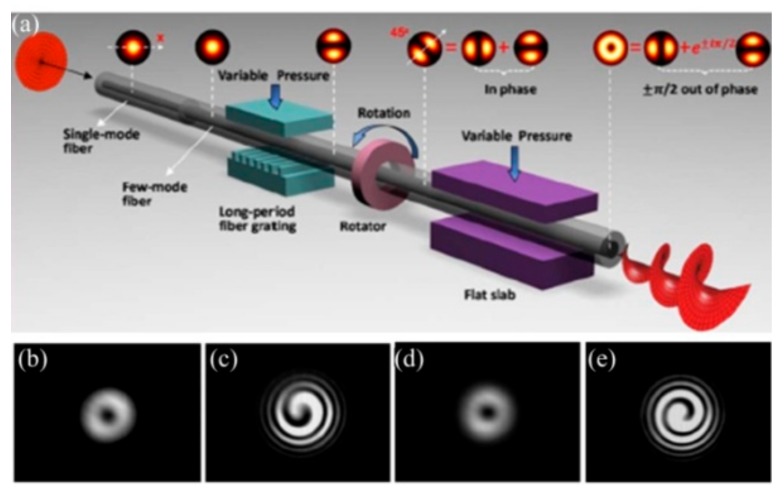
(**a**) Principle of the controllable all-fiber orbital angular momentum (OAM) mode converter. (**b**,**d**) intensity; and (**c**,**e**) interference pattern of the generated OAM_±1_ modes [51].

As shown in Figure 23, Li et al. proposed a controllable all-fiber OAM mode converter, consisting of a two-mode fiber (TMF), a mechanical-induced LPFG, a mechanical rotator, metal flat slabs, and a polarization controller [51].The mechanical-induced SLPFG converted the core mode to the high-order modes, and then a specific relative phase difference was achieved in combination of the rotator with flat slabs. By adjusting (stressing, rotating, and twisting) the mechanical device, an input LP_01_ mode from the SMF could be selectively converted to the LP_11a_, LP_11b_, OAM_−1_, or OAM_+1_ modes at the output terminal of the TMF.

**Figure 24:**
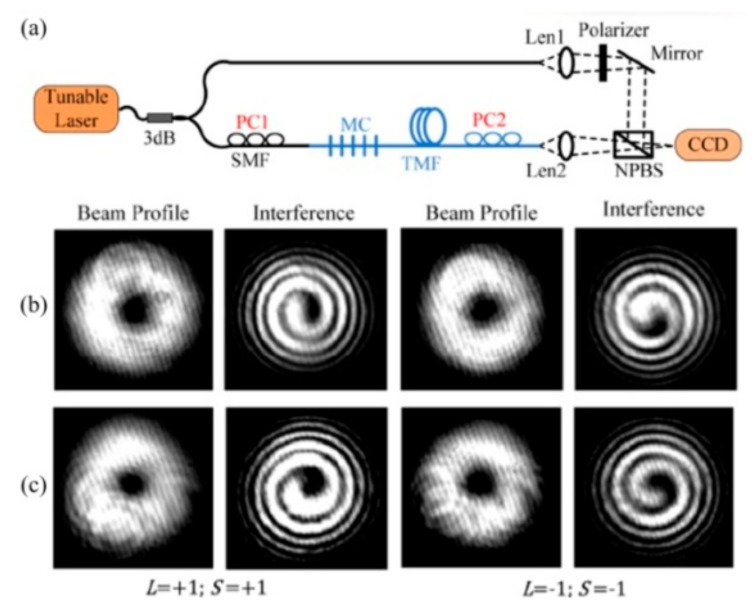
(**a**) Experimental setup used to excite the OAM modes; beam profile and interference patterns of the generated OAM modes using (**b**) SLPFG and (**c**) tilted SLPFG, respectively [52].

To enhance the stability of the OAM mode converter, Zhao et al. employed permanent CO_2_-laser-inscribed uniform and tilted SLPFGs in the TMF to be used as an OAM mode converter [52]. The mode conversion efficiency of the fabricated SLPFGs between LP_01_ mode and LP_11_ mode could be up to 99%. Moreover, the bandwidth and conversion efficiency of the tilted SLPFG, i.e., OAM mode converter, could be tuned by adjusting the fabrication tilt angle. As shown in Figure 24, two orthogonal vector modes (the HE_21_^even^ and HE_21_^odd^ modes) could be successfully generated by adjusting the polarization controllers in the uniform and tilted SLPFGs, thus generating the corresponding OAM modes at the resonant wavelength.

**Figure 25:**
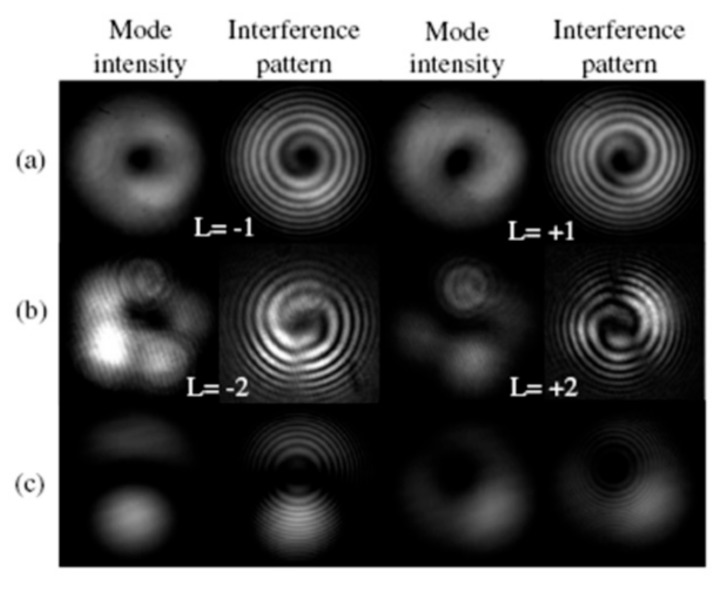
The generated OAM_±1_ and OAM_±2_ modes by using the three single SLPFG mode converters: (**a**) LP_01_−LP_11_; (**b**) LP_01_−LP_21_; and (**c**) LP_01_−LP_02_ [53].

Subsequently, the CO_2_-laser-inscribed SLPFGs in the FMF are also employed to generate the OAM_±1_ and OAM_±2_ modes [53], as shown in Figure 25. A single SLPFG could be used to convert the LP_01_ mode to LP_11_, LP_21_, and LP_02_ modes with a conversion efficiency of 99%. Moreover, an optimized LP_01_−LP_21_ mode converter with a maximum conversion efficiency of ~99.5% can be obtained by use of cascaded SLPFGs with different grating pitches. The low- and high-order OAM modes, i.e., OAM_±1_ and OAM_±2_ modes, are experimentally generated using the CO_2_-laser-inscribed SLPFGs in the FMF.

**Figure 26:**
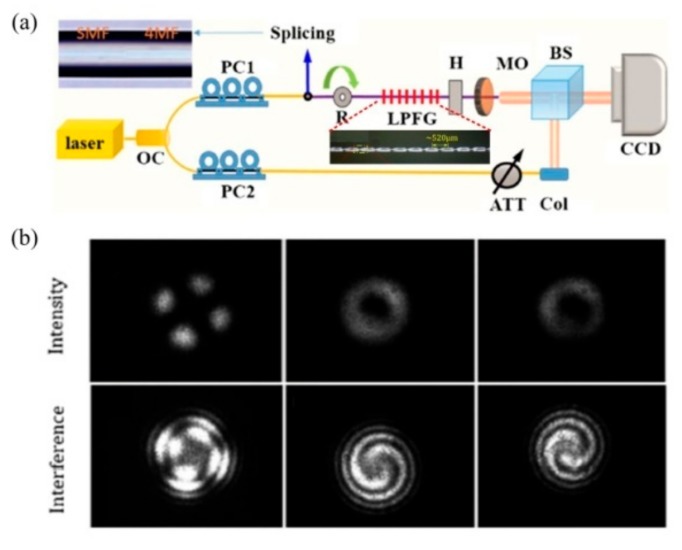
(**a**) Experimental setup for generating and detecting the OAM modes; (**b**) intensity profiles and interference patterns of the generated OAM_±2_ modes by only using one strong modulated SLPFG in FMF [54].

A single CO_2_-laser-induced SLPFG with strong periodic deformation inscribed in the FMF is used to directly convert the LP_01_ mode to an LP_21_ core mode. Then, a high-order OAM mode converter, i.e., OAM_±2_, via twisting a strong modulated SLPFG written in a FMF, was proposed and experimentally demonstrated [54]. As shown in Figure 26, we can directly convert the LP_01_ to LP_21_ with a conversion efficiency of 99.7% and then transform the LP_21_ mode into OAM_±2_ by adjusting the polarization controller and rotator. It is the first time that OAM_±__2_ modes were generated by only one SLPFG in FMF.

**Figure 27:**
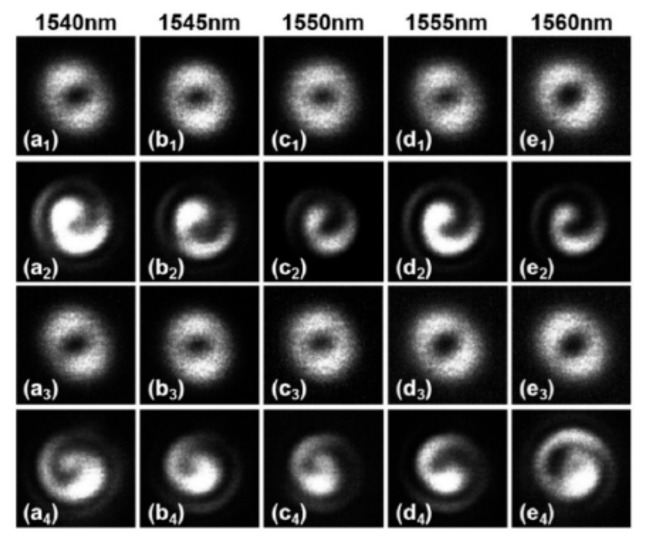
Mode intensities (**a_1_**–**e_1_**) and (**a_3_**–**e_3_**), interference patterns (**a_2_**–**e_2_**) and (**a_4_**–**e_4_**) of the generated OAM_±1_ modes via acoustically-induced fiber grating (AIFG) at different resonant wavelengths [55].

Zhang et al. realized the generation of the optical vortex with a wavelength tenability by using an acoustically-induced fiber grating (AIFG) [55]. As shown in Figure 27, the OAM mode could be generated from 1540 to 1560 nm by dynamically adjusting the frequency of the driving signal, i.e., radio frequency signal. The left- and right-handed circular polarization fundamental mode could be converted to the OAM_+1_ and OAM_−1_ modes, respectively, in the TMF using the AIFG with a conversion efficiency of ~95%.

**Figure 28:**
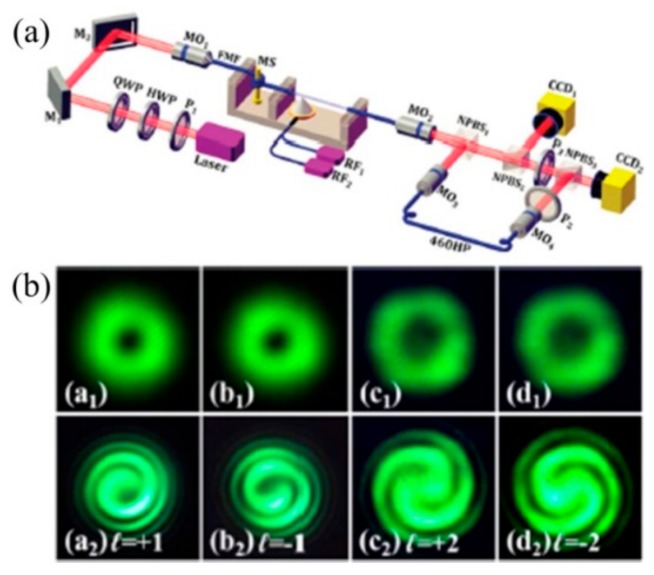
(**a**) Experimental setup for generating the OAM mode by using the cascaded AIFG; (**b**) beam profiles and interference patterns of the VM_11_^±^ and VM_21_^±^ [56].

Then, a high-order OAM mode converter, i.e., OAM_±2_ mode, was proposed by using the cascaded AIFG in the few-mode fiber [56]. As shown in Figure 28, the first AIFG was used to convert the circular polarization core mode to OAM_±1_, and then to convert the OAM_±1_ mode to OAM_±2_ mode through the second AIFG. In the experiment, the two AIFGs were driven by two different radio frequencies. 

**Figure 29:**
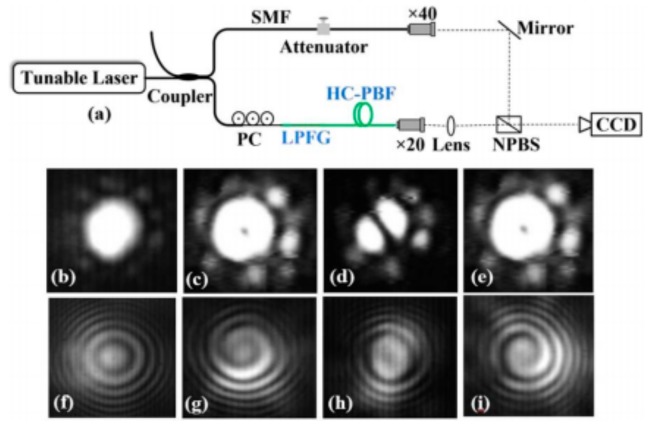
(**a**) Schematic diagrams for generating the OAM modes; (**b**–**e**) mode intensities and (**f**–**i**) interference patterns of the generated OAM modes [50].

In addition, the OAM modes based on SLPFG in air-core PBF have been obtained by superposing vector modes HE_21_^e,o^, because the effective refractive index separation of the second group of vector modes is around 10^–4^. Moreover, as shown in Figure 29, the phased delay between HE_21_^e^ and HE_21_^o^ has been adjusted by changing the polarization states of the input fundamental mode, and the topology charge of OAM has been tuned from −1 to +1 as well [50].

### 3.2. OAM Mode Converters Based on HLPFGs

OAM mode converters based on above-mentioned LPFGs require external devices, e.g., rotators, polarizers, and polarization controllers, to regulate the polarization direction or phase difference, which gives rise to a complex system. Then, the OAM modes could be generated only at specific phase difference. Wong et al. demonstrated, for the first time, the excitation of OAM modes in a continuously twisted solid-core PCF, but no OAM modes were experimentally observed due to a weak coupling [18].Then, Xi et al. studied the properties of a continuously twisted PCF with a novel three-bladed core that preserves the chirality of OAM modes of the same order, i.e., it inhibits scattering between an order +1 mode to an order –1 mode [20]. This is of potential interest for increasing channel capacity in optical telecommunications.

**Figure 30:**
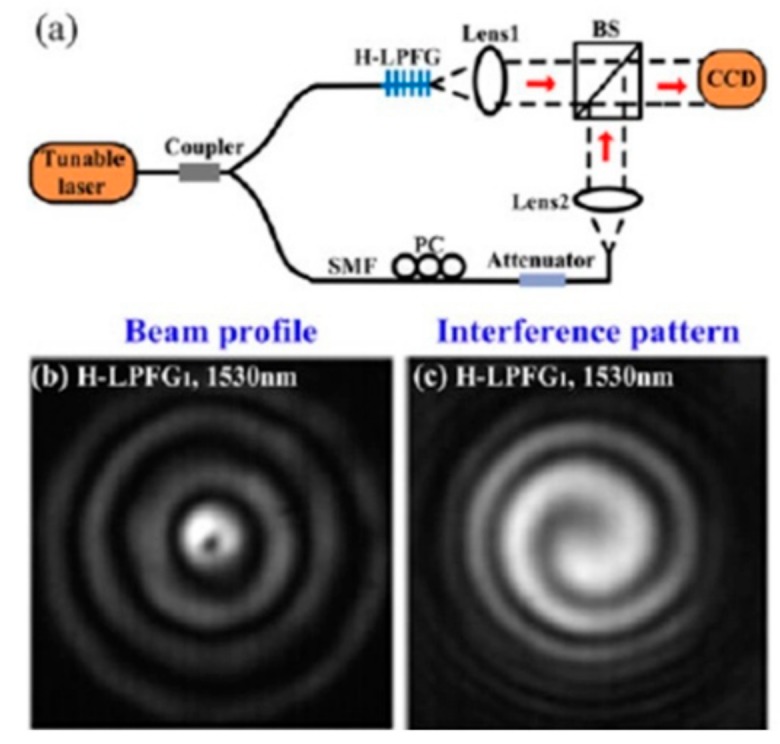
(**a**) Schematic diagram of experimental setup for detecting the generated OAM modes; (**b**,**c**) beam profile and interference pattern of the generated OAM_+1_ mode by the HLPFG in SMF at the resonant wavelength [13].

A novel all-fiber low-order OAM mode convertor based on HLPFG in SMF is demonstrated in Figure 30 by means of hydrogen–oxygen flame heating technique [13]. The purity and conversion efficiency of the generated OAM_+1_ mode are 91% and 87%, respectively. Moreover, the resonant wavelength of the HLPFGs, i.e., OAM mode converter, could be adjusted by changing the twist rate. In other words, the OAM_+1_ mode in the HLPFG could be generated within a large wavelength region using the HLPFG in SMF.

**Figure 31:**
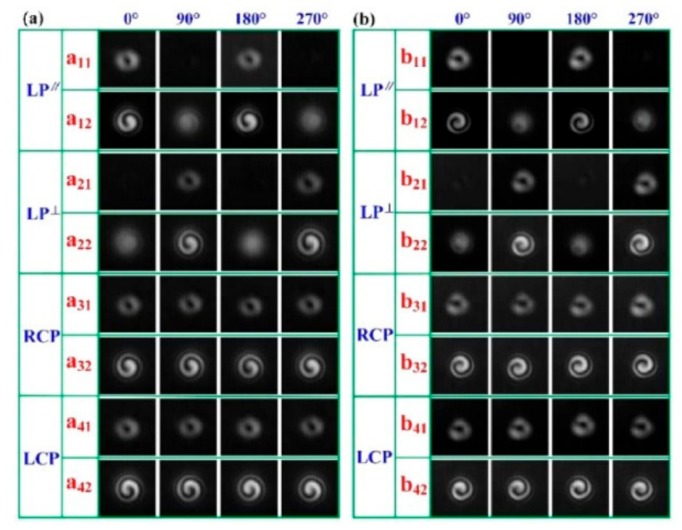
Beam profiles and interference patterns of the generated (**a**) OAM_+1_ modes and (**b**) OAM_−1_ modes by using right-handed and left-handed HLPFG in TMF, respectively [17].

Then, the authors have demonstrated a polarization independent OAM_±1_ mode converter based on HLPFG in TMF [17]. As shown in Figure 31, the OAM_+1_ and OAM_−1_ modes could be generated by the right- and left-handed HLPFG at different polarization states of the input light, indicating that the OAM mode converter based on HLPFG in TMF is polarization independent.

**Figure 32:**
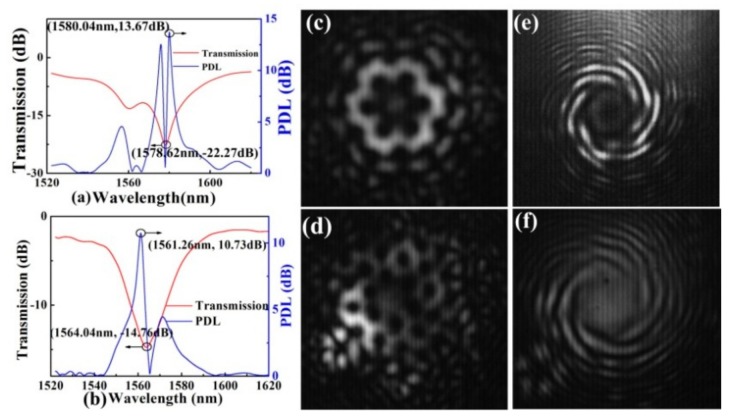
Transmission spectrum of the (**a**) Dip_1_ (**b**) and Dip_2_; (**c**,**d**) measured beam profiles and (**e**,**f**) interference patterns of the Dip_1_ and Dip2 in the HLPFG, i.e., OAM_+6_ and OAM_+5_ modes generated by the HLPFG in the PCF [14].

A special twisted three-bladed core PCF has generated an OAM_±1_ mode [20]. In-fiber high-order OAM mode converters could be used to achieve a higher data transmission capacity in an all-fiber optical communication system. A high-order OAM mode converter, i.e., OAM_+6_ and OAM_+5_, is achieved by a HLPFG in the PCF. As shown in Figure 32, the resonance dips, i.e., Dip_1_ and Dip_2_, of the HLPFG generated OAM_+6_ and OAM_+5_ modes [14]. Compared with [18], the reason for the successful observation of the generated OAM mode is a higher attenuation, i.e., –20 dB. Moreover, the resonant wavelength and attenuation of the HLPFG are determined by the twist rate (i.e., grating pitch) and coupling length.

**Figure 33:**
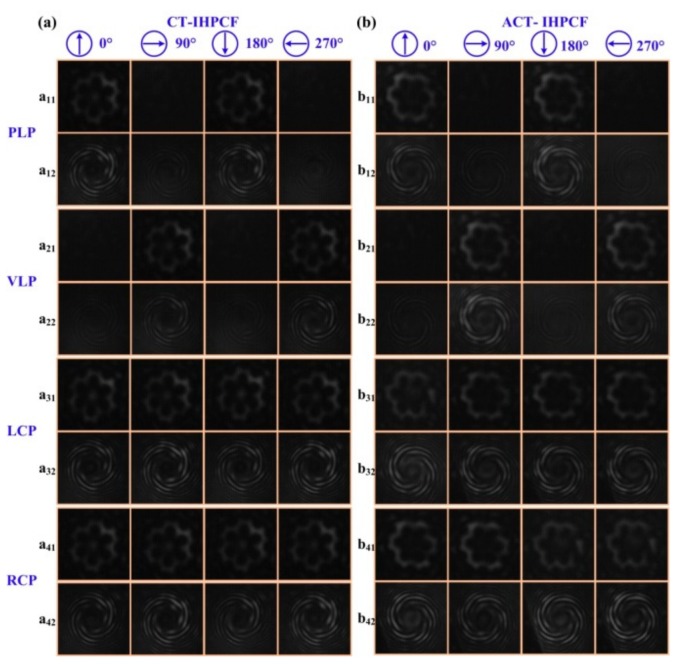
OAM_±6_ modes generated by the (**a**) CT- and (**b**) ACT-IHLPFG in the PCF at different polarization states of the input light [15].

Furthermore, as shown in Figure 33, a high-order twist-direction-dependent OAM_±6_ mode converter was achieved by an IHLPFG in the PCF [15]. Compared with un-inflated HLPFG in the PCF, the OAM mode converter based on IHLPFG generated a high-quality OAM_±6_ mode due to a perfect transmission dip without distinct splits. In addition, the order of the generated OAM modes was determined by the twist direction i.e., CT- and ACT-IHLPFG. Moreover, the polarization state of the input light, i.e., parallel linearly polarized (PLP), vertical linearly polarized (VLP), left circularly polarized (LCP) and the right circularly polarized (RCP) light has no effect on the order of the generated OAM mode.

## 4. Strain Sensors

The resonant wavelength will shift when the strain is applied to the LPFG, resulting from its stretched helical pitch or periodic micro-bends [25]. As a result, the LPFGs could be used as strain sensors.

A temperature-independent strain SLPFG sensor is fabricated by carving grooves periodically along a twisted SMF with high-frequency CO_2_ laser. The separation change between the two split peaks of SLPFGs exhibited a high strain sensitivity of about 106.7 pm/με [61]. Moreover, using a phase-shifted SLPFG fabricated by CO_2_ laser beam, a temperature-insensitive sensor that allows for large axial strain measurement with a value up to 7600 με is demonstrated [62]. Ren et al. developed a strain sensor with a sensitivity of 1.2 pm/με based on a microtapered SLPFG fabricated by periodically tapering a SMF with CO_2_ laser heating source [63]. Furthermore, two peaks of the novel high-sensitivity strain sensor fabricated by weak power modulation of CO_2_ laser exposure on a tapered LPFG exhibited sensitivities of 1.82 and 8.17 pm/με, respectively [64].

**Figure 34:**
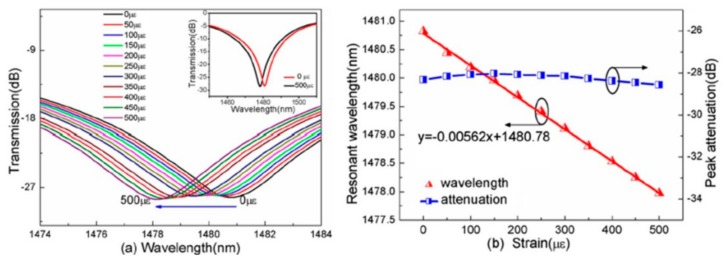
(**a**) Transmission spectrum evolution of the strain sensor based on ILPFG with a sensitivity of –5.62 pm/με; (**b**) measured resonant wavelength and peak attenuation as a function of the strain [48].

A SLPFG strain sensor based on PCF with a high strain sensitivity of −7.6 pm/με and a low temperature sensitivity of 3.91 pm/°C is achieved by use of a focused CO_2_ laser beam carving periodic grooves on the fiber [57]. Such a strain sensor can effectively reduce the cross-sensitivity between strain and temperature, and the temperature-induced strain measurement error is only 0.5 με/°C in the case of no temperature compensation. Another high-sensitivity strain sensor based on an inflated SLPFG (I-SLPFG) was fabricated by use of the pressure-assisted CO_2_ laser beam scanning technique to periodically inflate air holes of the PCF. As shown in Figure 34, such periodic inflations of the holes enhanced the strain sensitivity of the ISLPFG to –5.62 pm/με [48]. In addition, after high temperature annealing, the I-SLPFG demonstrated a good repeatability and stability of temperature response, and a sensitivity of 11.92 pm/°C was achieved.

Strain sensors based on HLPFG have also been developed. The strain sensitivities of HLPFGs fabricated by twisting SMF during CO_2_ laser irradiation are characterized in terms of both spectral shift and transmission power variation. Results show that the resonant wavelength shift sensitivity and transmission power sensitivity are –1.1 pm/με and 2.2 × 10^–4^ dB/με, respectively [58]. In addition, a novel strain sensor based on two successively cascaded HLPFGs exhibits a sensitivity of −0.185 pm/με [59]. A highly sensitive strain sensor with a sensitivity of –61.8 pm/με based on helical structure-assisted Mach–Zehnder interference in an all-solid heterogeneous multi-core fiber was proposed and experimentally demonstrated [60]. Xi et al. investigated a strain sensor with a sensitivity of –1.18 pm/με based on HLPFG in the PCF, and the analysis showed that the tension-induced shift in resonance wavelength is determined both by the photoelastic effect and the change in twist rate [19]. The author also proposed a strain sensor with similar sensitivities of –3.20 and –3.18 pm/με based on the HLPFG and I-HLPFG in the PCF [16]. 

## 5. Pressure Sensors

Various grating-based pressure sensors are of great interest owing to their easy fabrication, compact size, and robustness. The resonant wavelength of the LPFG would be observed by the red or blue shifts with the change of pressure (transverse load).

A compact reflective type of pressure sensor based on tapered SLPFGs fabricated by means of a computer-assisted precision arc-discharge apparatus in SMF had a sensitivity of 0.51 pm/MPa [65]. Then, a pressure sensor based on tapered SLPFGs in PCF displayed a pressure sensitivity of 1.12 pm/MPa [66]. Subsequently, Zhong et al. demonstrated an I-LPFG in the PCF by use of the pressure-assisted CO_2_ laser beam-scanning technique to periodically inflate air holes of a PCF along the fiber axis. Such an I-SLPFG exhibited a pressure sensitivity of 1.68 nm/MPa, which is higher than the SLPFG without inflation. Moreover, the I-SLPFG exhibited a low temperature sensitivity, i.e., 3.1 pm/°C, due to the inherent pure silica material, resulting in a low 1.8 Kpa/°C cross-sensitivity in the case of no temperature compensation [29].

Tang et al. reported a gas pressure sensor with periodic collapses of air holes in an air-core PBF fabricated by use of focused CO_2_ laser beam [30]. The gas pressure sensor exhibited a pressure sensitivity of –137 pm/MPa due to the gas-pressure-induced stress concentration at the collapse region of the LPFG in PBF. Then, an improved gas pressure sensor constructed by a short hollow silica tube segment with a SLPFG in PBF was proposed [31]. A micro-channel in the middle of the hollow silica tube was introduced by use of the femtosecond laser technique to allow the air-core of the hollow core PBF to be exposed to the external gas surroundings. The gas pressure sensitivity of the sensor was increased to –1.3 nm/MPa. Moreover, the temperature sensitivity of the gas-pressure sensor was as low as 5.3 pm/°C.

**Figure 35:**
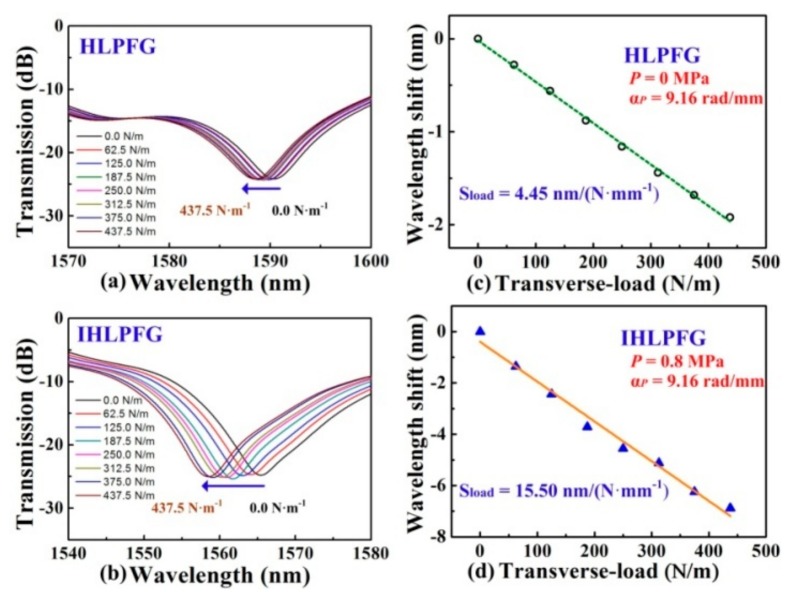
Transmission spectrum evolution of the (**a**) HLPFG and (**b**) I-HLPFG in PCF with air-hole diameters of 2.9 and 3.6 μm, respectively; measured resonant wavelength shift of the (**c**) HLPFG and (**d**) I-HLPFG as a function of the transverse load [16].

Furthermore, the HLPFG and I-HLPFG with different air-hole diameters, i.e., 2.9 and 3.6 μm, could be used as a pressure senor [16]. The transverse-load sensitivity, i.e., 15.50 nm/(N·mm^−1^), of the IHLPFG with an air-hole diameter of 3.6 μm was higher than the transverse-load sensitivity of the HLPFG with an air-hole diameter of 2.9 μm, i.e., 4.45 nm/(N·mm^−1^), as shown in Figure 35. In other words, we could greatly enhance the transverse-load sensitivity by enlarging the diameter of the air holes in the I-HLPFG.

## 6. Torsion Sensors

Torsion sensors are widely used in various applications due to their high torsion sensitivity. Various types of optical fiber torsion sensors, such as corrugated SLPFGs [101], high-birefringence fibers [102], polarization-maintaining FBGs [103], Sagnac loops [104,105], and distributed FBGs [106] have been demonstrated. Unfortunately, the torsion directions, i.e., clockwise- or anticlockwise-twisted, could not be distinguished by these devices. Wang et al. reported that a CO_2_-laser-induced LPFG could not only measure the applied twist rate but also distinguish simultaneously the torsion direction, i.e., the resonant wavelength of the CO_2_-laser-induced SLPFG shifts linearly toward the longer wavelength under clockwise torsion, while the opposite process occurred under anticlockwise torsion [107,108].

**Figure 36:**
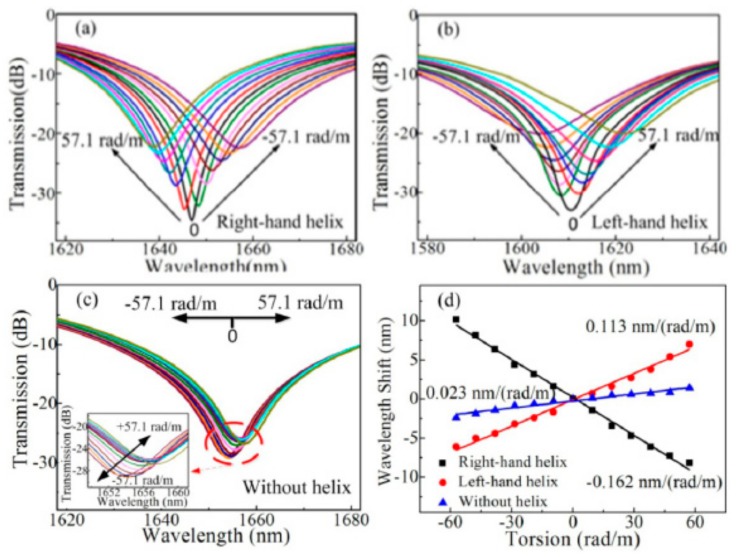
Resonant wavelength shifts for (**a**) right-hand; and (**b**) left-handed pre-twisted LPFG; and (**c**) conventional LPFG. (**d**) Measured resonant wavelength shift versus the applied torsion [46].

**Figure 37:**
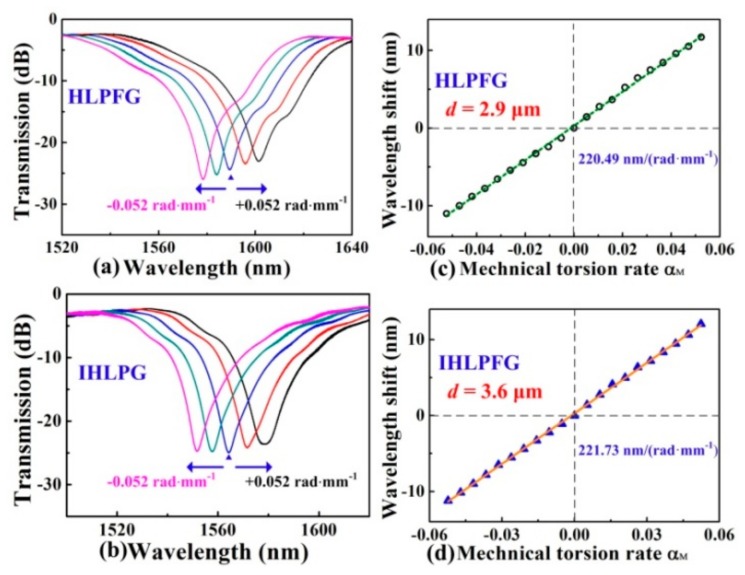
Transmission spectrum evolution of the (**a**) HLPFG and (**b**) I-HLPFG in PCF with air-hole diameters of 2.9 and 3.6 μm, respectively; measured resonant wavelength shift of the (**c**) HLPFG and (**d**) I-HLPFG as a function of the mechanical torsion [16].

A novel pre-twisted LPFG with periodic reserved screw-type deformations was developed to be a torsion sensor to distinguish not only the torsion angle but also the torsion direction [46]. As shown in Figure 36, the opposite resonant wavelength shifts with sensitivities of 160.4 and 113.0 nm/(rad·mm^−1^) were observed for the right- and left-helix pre-twisted LPFGs. The resonant wavelengths of the HLPFG in SMF fabricated by automatic arc discharge heating technique vary monotonically and linearly with a torsion sensitivity of –46.46 nm/(rad·mm^−1^) [37]. A type of few-period helically twisted all-solid PBF produces mechanical torsion sensitivities of 115.5 nm/(rad·mm^−1^) [38]. Moreover, as shown in Figure 37, the HLPFG and I-HLPFG in the PCF exhibit similar high torsion sensitivities, i.e., 220.49 and 221.73 nm/(rad·mm^−1^) [16]. According to the experimental results, we could distinguish the applied torsion direction by observing blue- or red-shift of the resonant wavelength.

In addition, a HLPFG in the multi-core fiber (MCF) fabricated by CO_2_ laser heating technology shows a torsion sensitivity up to 198 nm/(rad·mm^−1^) [77]. A directional torsion sensor fabricated by twisting the MCF based on the Mach–Zehnder interferometer exhibited a sensitivity of ~118.0 nm/(rad·mm^−1^) while the applied torsion changed from −17.094 rad/m to 15.669 rad/m [109]. 

## 7. Biochemical Sensors

The resonant wavelength or attenuation peak of the SLPFG is sensitive to the surrounding refractive index due to the cladding mode coupled from the fundamental light [67]. SLPFG sensors have drawn great attention in the field of refractive index sensing owing to the label-free, high sensitivity, and real-time measurement. Moreover, specific detections could be achieved by modifying different types of functional material on the surface of the SLPFGs. SLPFGs have been utilized in chemical and biomass applications such as the detection of specific biological molecules, composition or content of a gas, and corrosion in the steel.

**Figure 38:**
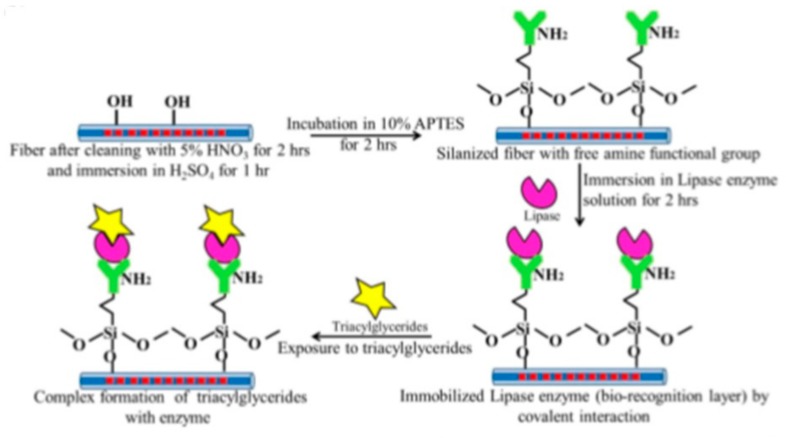
Schematic diagram of the layer-by-layer modification of SLPFG.

A chemical sensor coated with the functional material, i.e., incorporation of diphenyl siloxane and titanium cross-linker on the SLPFG, successfully realized the chemical differentiation of cyclohexane and xylene, where the detection limit is 300 ppm for the xylene vapor [68]. A stable sensor using SLPFG has been developed for the detection of triacylglycerides, where the lipase enzyme is immobilized on the fiber by means of covalent binding technique, as shown in Figure 38. The sensors showed a sensitivity of 0.5 nm/mM and a low detection limit of 17.71 mg/dL in human blood [69]. 

The detection of the composition or content of a gas is necessary in industry and healthcare. Hromadka et al. presented a SLPFG-based carbon dioxide (CO_2_) sensor by coated with HKUST-1, i.e., a material from the metal–organic frame on the fiber. The SLPFG coated with 40 layers HKUST-1 by means of layer-by-layer techniques was sensitive to the CO_2_ concentration between 2000 and 40,000 ppm with a detection limit of 401 ppm [70]. The methane sensor was fabricated by depositing the inclusion of cryptophane A in the styrene–acrylonitrile cladding on the SLPFGs, where the sensing film is sensitive to the adsorption of methane in the surroundings. The achieved sensor based on SLPFG exhibited a good sensitivity (~0.375 nm %^–1^) for the methane below 3.5 vol% [71]. 

SLPFG sensor coated with a layer of polyurethane and iron/silica nanoparticles was developed to detect the corrosion process of deformed steel bars. However, the experimental results show that the corrosion sensitivity would decrease significantly with the extension of the service time [72]. Then, a sensor based on SLPFG written in SMF by a transverse focused CO_2_ laser was demonstrated to monitor the corrosion process of carbon steel in a 3.5 wt% NaCl solution. The SLPFG was firstly deposited with a ~0.8-μm-thick Ag film, and then electroplated an outer ~20-μm-thick Fe–C film. In addition, the thickness of the Ag and Fe–C strongly affected the corrosion sensitivity and the lifecycle [73].

## 8. Conclusions

In conclusion, the recent progress in fabrications and applications of heating-induced LPFGs have been reviewed in this work. High-quality SLPFGs and HLPFGs in conventional glass fibers, PCFs, and PBFs could be obtained by use of three kinds of heating fabrication techniques, i.e., CO_2_ laser, arc discharge, and hydrogen–oxygen flame. Compared with SLPFG, HLPFG refers to a fiber where there exists a periodical helical structure such as a screw index-modulation along the fiber axis. Then, the OAM mode convertors based on SLPFGs and HLPFGs have been described. The OAM mode convertors based on SLPFGs require external devices, e.g., rotators, polarizers, polarization controllers, or piezo delay stages, to adjust the phase difference or polarization direction; while the HLPFGs could directly generate the OAM modes due to their helical structure. Heating-induced LPFGs have found promising sensing applications, such as strain, pressure, torsion, and biochemical sensors. The HLPFGs could distinguish the torsion direction by observing the red-shift or blue-shift of the resonant wavelength.
